# Compositional Stability of a Salivary Bacterial Population against Supragingival Microbiota Shift following Periodontal Therapy

**DOI:** 10.1371/journal.pone.0042806

**Published:** 2012-08-16

**Authors:** Wataru Yamanaka, Toru Takeshita, Yukie Shibata, Kazuki Matsuo, Nobuoki Eshima, Takeshi Yokoyama, Yoshihisa Yamashita

**Affiliations:** 1 Section of Preventive and Public Health Dentistry, Division of Oral Health, Growth and Development, Kyushu University Faculty of Dental Science, Fukuoka, Japan; 2 Section of Dental Anesthesiology, Division of Maxillofacial Diagnostic and Surgical Sciences, Kyushu University Faculty of Dental Science, Fukuoka, Japan; 3 Department of Biostatistics, Oita University Faculty of Medicine, Yufu City, Oita, Japan; University of Illinois, United States of America

## Abstract

Supragingival plaque is permanently in contact with saliva. However, the extent to which the microbiota contributes to the salivary bacterial population remains unclear. We compared the compositional shift in the salivary bacterial population with that in supragingival plaque following periodontal therapy. Samples were collected from 19 patients with periodontitis before and after periodontal therapy (mean sample collection interval, 25.8±2.6 months), and their bacterial composition was investigated using barcoded pyrosequencing analysis of the 16S rRNA gene. Phylogenetic community analysis using the UniFrac distance metric revealed that the overall bacterial community composition of saliva is distinct from that of supragingival plaque, both pre- and post-therapy. Temporal variation following therapy in the salivary bacterial population was significantly smaller than in the plaque microbiota, and the post-therapy saliva sample was significantly more similar to that pre-therapy from the same individual than to those from other subjects. Following periodontal therapy, microbial richness and biodiversity were significantly decreased in the plaque microbiota, but not in the salivary bacterial population. The operational taxonomic units whose relative abundances changed significantly after therapy were not common to the two microbiotae. These results reveal the compositional stability of salivary bacterial populations against shifts in the supragingival microbiota, suggesting that the effect of the supragingival plaque microbiota on salivary bacterial population composition is limited.

## Introduction

Saliva is a promising specimen for investigations of the oral environment [1]. Various components of the salivary bacterial population have been implicated in periodontal disease [2], halitosis [3], oral cancer [4], obesity [5], and pancreatic disease [6]. Saliva seems to reflect the overall oral microbial environment, which is composed of more than 700 indigenous bacterial species [7], [8]. A variety of architectures and environments exist in the oral cavity, such as the tooth surface, buccal and tongue mucosal surfaces, and subgingival crevices, each of which provides a different ecological niche. Therefore, a variety of characteristic microbiota are formed on each saliva-bathed oral surface. Recently, Mager et al. demonstrated that the salivary bacterial profile is closer to that of the mucosal surface than that of dental plaque using DNA–DNA hybridization targeted to 40 selected bacterial species [9]. This result was further supported by a 16S rRNA gene pyrosequencing study of three healthy subjects [10].

Saliva is often used clinically as an alternative to dental plaque. For example, dental caries activity assessment kits for detecting cariogenic bacteria often utilize saliva, even though the principal niche of these microorganisms must be dental plaque. The presence or absence of periodontal pathogens in saliva is also expected to be useful for the diagnosis of periodontitis [11]. Moreover, teeth cleaning is strongly recommended to prevent aspiration pneumonia [12], although the main cause of the condition is bacteria contained in aspirated saliva. These concepts are contradictory if the plaque microbiota has a limited effect on the salivary bacterial population. Therefore, it is important to clarify the source of the salivary bacterial population.

Patients with periodontal diseases are generally treated initially with nonsurgical periodontal therapy comprising the mechanical removal of supra- and subgingival plaque and calculus by periodontal scaling and professional tooth cleaning in addition to patient education, training in personal oral hygiene, and counseling on the control of risk factors, e.g., smoking and diabetes mellitus. Although some patients are also treated with antibiotics or surgical periodontal treatment based on the clinical evaluation of the individual patients' response to the initial therapy, most patients move to supportive maintenance therapy after the completion of nonsurgical periodontal treatment.

In this study, we collected supragingival plaque and saliva from 19 periodontal patients during pre- and post-periodontal therapy without surgical treatment and determined the bacterial compositions by barcoded pyrosequencing analysis of the 16S rRNA gene. We observed a compositional shift in the salivary bacterial population after periodontal therapeutic intervention, which seemed to affect the supragingival plaque microbiota both qualitatively and quantitatively. The objective of this study was to clarify the extent to which the supragingival plaque microbiota influences the salivary bacterial population through dynamic analysis of the population shift in stimulated saliva and supragingival plaque.

## Results

### Subjects' clinical characteristics

In this study, 19 patients with periodontitis visiting a dental clinic (seven women and twelve men, aged 35–73 years) were recruited (Table 1). Supragingival plaque and saliva samples were collected from each subject at the first visit and approximately 2 years later (mean sample collection interval, 25.8±2.6 months). Until the time of the second sample collection, all subjects had completed periodontal therapy and had been in supportive therapy with maintenance care. The clinical periodontal condition of every subject improved after therapeutic intervention (Table 1).

**Table 1:** Clinical parameters of the 19 subjects enrolled in this study.

				Percentage of periodontal pocket sites				
				Pre-therapy		Post-therapy		
Subject number	Age (yr)	Sex	Number of teeth	>4 mm	>7 mm	>4 mm	>7 mm	Interval (months)
1	49	Male	28	43.5	17.3	19.1	1.2	30
2	46	Male	28	77.7	29.6	23.5	3.1	28
3	58	Male	22	22	0.8	8.3	1.5	29
4	73	Male	23	41.3	1.4	13.7	1.4	27
5	73	Male	26	49.4	5.8	31.4	3.8	26
6	64	Male	28	35.7	1.8	23.6	0	27
7	58	Male	28	69.1	16.7	33.3	1.8	29
8	51	Female	25	72.7	12.7	8.4	1.3	27
9	56	Male	27	37.6	11.7	17.9	1.9	28
10	64	Female	32	4.2	0	2.6	0	27
11	57	Female	29	41.3	0.6	3.1	0	27
12	35	Male	19	28.1	1.8	17.6	0	25
13	72	Male	27	31.5	0	16	0	26
14	48	Female	30	39.4	6.1	15.5	1.1	23
15	59	Male	21	21.4	7.1	9.6	5.6	23
16	63	Male	20	85.8	33.3	60.9	11.7	23
17	57	Female	28	36.3	7.7	18.5	3.6	24
18	43	Female	25	49.3	13.3	16.7	2.8	22
19	59	Female	27	38.2	11.7	21.8	5.8	20

### Overall bacterial community composition in supragingival plaque and saliva

The bacterial composition of supragingival plaque and saliva was investigated using barcoded pyrosequencing analysis of the 16S rRNA gene. We determined 458,721 bacterial 16S rRNA gene sequences (containing the V1–V2 region), of which 255,062 passed quality control. A data set of an average of 3,356 sequences per sample with an average length of 343±24 bases (Table 2) was generated. The sequences were assigned to 8,497 species-level operational taxonomic units (OTUs) using a cutoff distance of 0.03.

**Table 2:** Number of sequence reads that passed quality filtering.

	Supragingival plaque		Saliva	
Subject number	Pre-therapy	Post-therapy	Pre-therapy	Post-therapy
1	3372	2592	2583	3331
2	3745	4221	2914	3371
3	3375	2604	2991	3096
4	3014	3762	3329	3689
5	3315	3918	2724	3008
6	3594	3587	2708	2792
7	3906	3014	3170	3307
8	3127	2378	2979	3119
9	3885	2645	3157	3883
10	3329	2574	3053	3180
11	4468	2991	4320	3044
12	3995	3257	3196	3622
13	3636	2810	3353	3795
14	3399	3830	3415	3301
15	3457	3138	3216	4021
16	3105	3514	3173	4165
17	4053	3316	3284	3042
18	3898	3893	3765	3724
19	3402	3715	3669	2739
Mean ± SD	3582±337	3210±403	3250±556	3380±411

**Figure 1:**
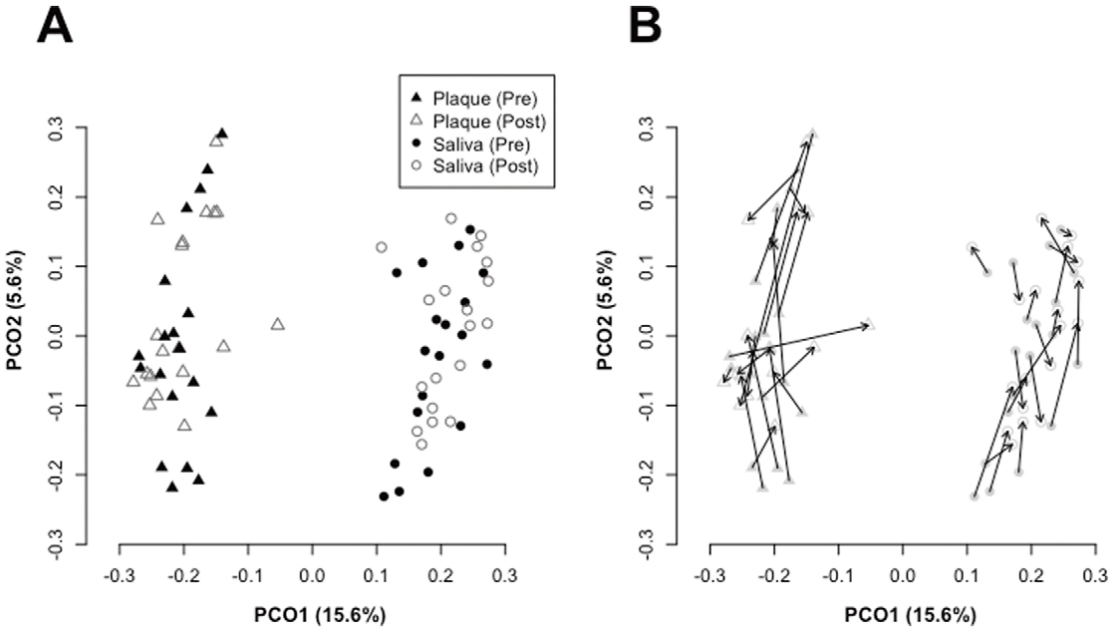
Principal coordinate analysis (PCoA) plot. (A) Similarity relations among the 76 bacterial community samples. Plots were generated using the unweighted UniFrac metric. These two components explain 21.2% of the variance. (B) Microbiota shift after periodontal therapy. Pre- and post-therapy supragingival plaque or saliva samples from the same subjects are connected by arrows.

The overall bacterial community composition was compared using UniFrac, which is a phylogeny-based distance metric ranging from 0 (identical bacterial communities) to 1 (totally different). A principal coordinate analysis (PCoA) plot based on unweighted UniFrac values revealed strong clustering of plaque and saliva samples, indicating that the composition of salivary bacterial populations was distinct from that of supragingival plaque microbiota both pre- and post-therapy (Figure 1A). The temporal variation following periodontal therapy was smaller than the compositional difference between the two bacterial communities.

**Figure 2:**
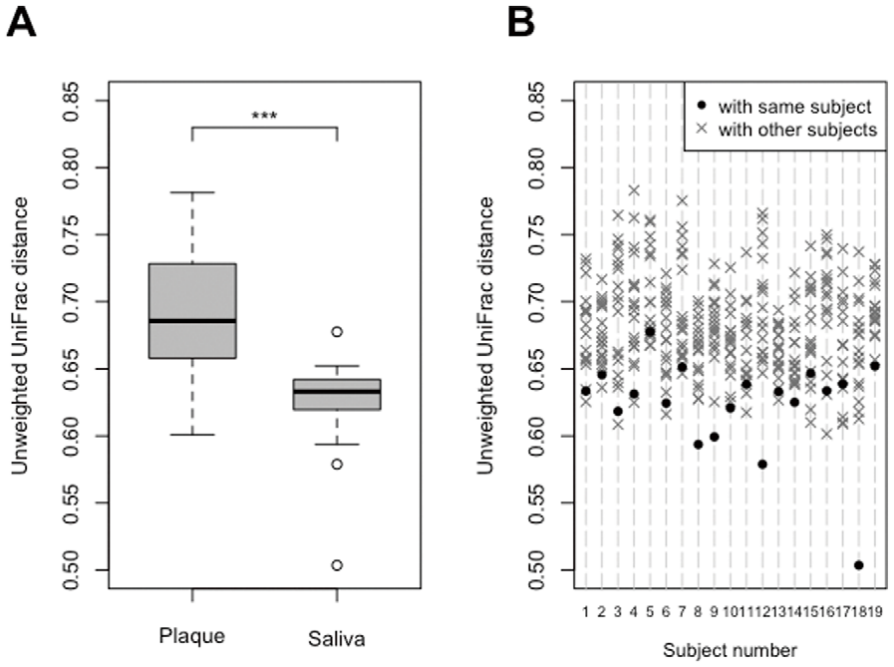
Unweighted UniFrac distance between pre- and post-therapy samples. (A) Degree of temporal variation in supragingival plaque microbiota and salivary bacterial population. Significant differences between the supragingival plaque microbiota and salivary bacterial population were assessed using paired t-tests. ***P<0.001. (B) Unweighted UniFrac distance between pre-and post-therapy saliva samples. UniFrac distance of the combination of pre- and post-therapy of the same individual (•) and those of the other 18 individuals (×) were plotted for each subject. A significant difference was observed between the UniFrac distance of the combination of pre- and post-therapy of the same individual and those of the other 18 individuals by Student's *t*-test (*P*<0.001).

The degree of temporal variation in overall bacterial community composition based on UniFrac distance was compared between supragingival plaque and saliva (Figure 1B). The difference in microbiota composition between pre- and post-therapy supragingival plaque samples was significantly larger than that in saliva (Figure 2A). Additionally, post-therapy saliva samples were significantly more similar to those pre-therapy from the same individual than pre-therapy samples from other subjects (Figure 2B), implying that the overall microbiota composition of pre-therapy saliva was well conserved after periodontal therapy. This was also confirmed in the comparison using weighted UniFrac values (Figure S1A–C), although the stability of the salivary bacterial community was slightly weaker when also considering abundance information (Figure S1C).

### Differences in microbiota composition between the supragingival plaque microbiota and salivary bacterial population

**Figure 3:**
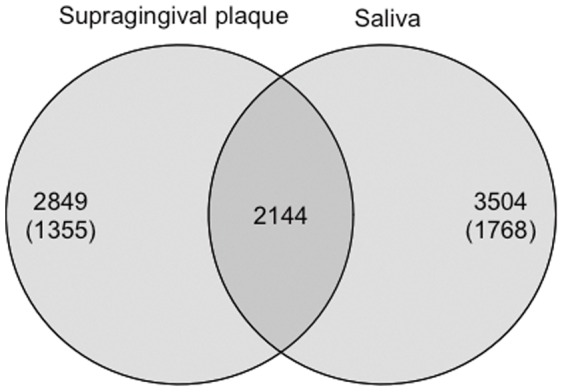
Venn diagram of the overlap between supragingival plaque observed operational taxonomic units (OTUs) vs. saliva observed OTUs. The numbers of singleton OTUs, detected from only one read from one subject, are shown in parentheses.

Compositional differences between supragingival plaque and saliva were observed in the microbial richness, diversity, and relative abundances of each taxon. The microbial richness estimated by the Chao I and ACE indices and the biodiversity assessed by the Shannon index were significantly higher in salivary bacterial populations than in the supragingival plaque microbiota (Table 3). Of 8,497 OTUs, 2,144 were commonly identified in both microbiota and their relative abundances constituted approximately 80% of the microbiota of each individual (data not shown). On the other hand, 2,849 and 3,504 OTUs were specifically detected in supragingival plaque and saliva samples, respectively, although half of them were singleton OTUs (Figure 3). The community membership, especially minor components, were substantially different between the two microbiota.

**Table 3:** Sequence diversity in each sample.

	Pre-therapy		Post-therapy	
	Plaque	Saliva	Plaque	Saliva
Number of OTUs	549±101	695±95^a^	442±77^b^	697±97^a^
Chao I	1039±217	1376±200^a^	828±151^b^	1352±234^a^
ACE	1059±210	1420±213^a^	845±157^b^	1402±235^a^
Shannon index	4.8±0.4	5.3±0.2^a^	4.5±0.4^b^	5.3±0.2^a^

Statistical differences were calculated using paired *t*-tests.

a Significantly greater than supragingival plaque samples obtained at the same time.

b Significantly less than pre-therapy samples.

The majority of sequences in both the supragingival plaque microbiota and salivary bacterial population were assigned to five bacterial phyla (Actinobacteria, Bacteroidetes, Fusobacteria, Firmicutes, and Proteobacteria; Figure S2). TM7, Spirochaetes, SR1, Tenericutes, Synergistetes, and Cyanobacteria were also identified from multiple subjects but in much lower proportions. Their relative abundances in plaque and saliva samples differed significantly (Table S1).

**Figure 4:**
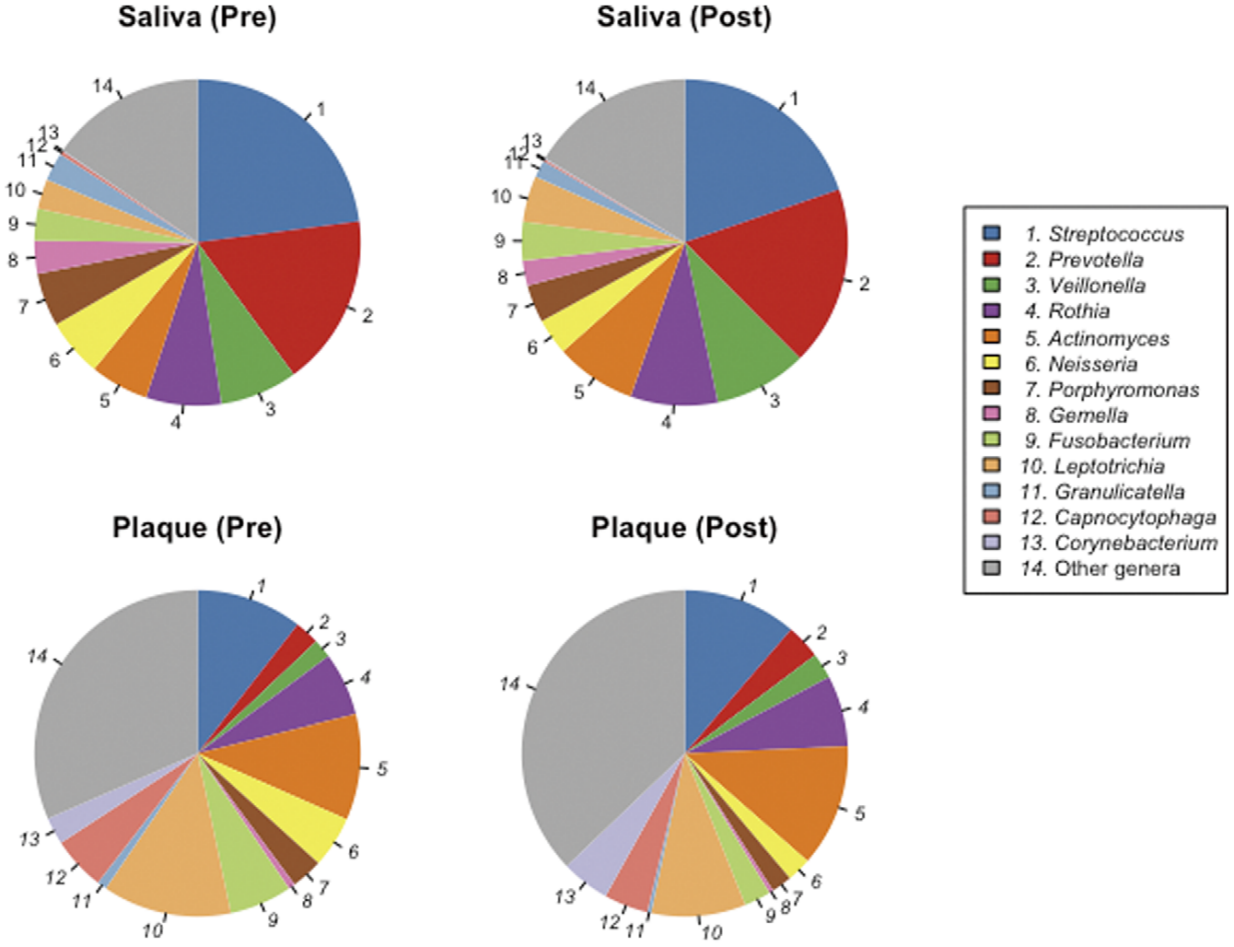
Mean genus abundances in the supragingival plaque microbiota and salivary bacterial population before and after periodontal therapy. Only 13 genera commonly detected in supragingival plaque or saliva samples are shown.

In total, 92 bacterial genera were identified in our data set. They constituted 84.1±8.3% (mean ± SD) of each bacterial population; the remaining unclassified sequences were assigned to 49 upper-level taxa. The genera abundance distribution in the microbiota also greatly differed between the two bacterial communities (Figure 4 and Table S2). Whereas 16 bacterial genera in saliva, including Streptococcus, Prevotella, and Veillonella, were significantly more predominant than those in the plaque microbiota, 22 genera in the plaque microbiota were present in significantly higher proportions than those in the salivary bacterial population. More specifically, Capnocytophaga and Corynebacterium, common dominant members of the plaque microbiota, represented only a small minority of the salivary bacterial population (Figure 4 and Table S2).

These differences in bacterial composition between saliva and plaque are consistent with previous studies [9], [10], [13], [14]. In particular, the results obtained by Keijser et al. [13] and Ling et al. [14] are comparable to our data, considering the use of pyrosequencing and the number of subjects. Although our results are consistent with the data reported by Keijser et al. [13], note that in their study, the 16S rRNA gene amplicons (lacking a barcode sequence) of each subject were mixed before pyrosequencing individual deviations were excluded. Ling et al. [14] evaluated microbiota in saliva and supragingival plaques from 60 children aged 3–6 years old by pyrosequencing and demonstrated a predominance in saliva of *Streptococcus*, *Prevotella*, and *Rothia* and in supragingival plaque, a predominance of *Neisseria*, *Actinomyces*, *Leptotrichia*, and *Thiomonas*. These observations were partially consistent with our results and the discrepancy between the two studies might have derived from the use of subjects of different ages; our subjects were aged 35–73 years.

### Differences in compositional shift between the supragingival plaque microbiota and salivary bacterial population

Temporal variation appeared in the supragingival plaque microbiota in terms of microbial richness and diversity. The number of OTUs detected, the microbial richness estimated by the Chao I and ACE indices and the biodiversity assessed by the Shannon index were also significantly lower in the plaque microbiota following periodontal therapy (Table 3). In contrast, no such decrease occurred in the salivary bacterial population (Table 3). When singleton and doubleton OTUs were excluded to eliminate transient bacteria from the analysis, the number of disappeared OTUs in supragingival plaque microbiota was significantly greater than that of salivary bacterial populations (Table 4). The greater numbers of disappeared OTUs compared with those of appeared OTUs in plaque microbiota were consistent with the decrease of microbial richness. Of the disappeared OTUs, those corresponding to bacterial genera such as *Tannerella*, *Porphyromonas*, *Leptotrichia*, and *Capnocytophaga* commonly disappeared after therapy from the supragingival plaque microbiota of more than six subjects, whereas their disappearance was observed in the salivary bacterial populations of only a few subjects (Table 5).

**Table 4:** Number of operational taxonomic units (OTUs) that either disappeared from or appeared in the microbiota of each subject after periodontal therapy.

	Plaque	Saliva	*P*-value
Number of disappeared OTUs	94±27	51±19	<0.001
Number of appeared OTUs	54±22	65±23	0.22

The OTUs detected in pre-therapy samples with more than two reads but not in post-therapy samples were defined as “disappeared OTUs” and the OTUs that were not detected in pre-therapy samples but detected in post-therapy samples with more than two reads were defined as “appeared OTUs.”

Statistical differences were calculated using paired *t*-tests.

**Table 5:** Taxonomic information for the operational taxonomic units (OTUs) that disappeared from supragingival plaque microbiota after periodontal therapy.

		Number of subjects with OTU dissipation	
OTU number	Taxonomic information	Plaque	Saliva
2898	*Tannerella*	10	0
2735	*Tannerella*	9	0
1827	Order *Clostridiales*	8	0
1212	*Porphyromonas*	7	1
2099	Family *Neisseriaceae*	7	0
5585	*Leptotrichia*	7	1
1081	*Capnocytophaga*	6	0
1173	*Capnocytophaga*	6	0
1792	*Actinomyces*	6	1
1891	*Capnocytophaga*	6	0
1922	*Actinomyces*	6	0
2757	*Leptotrichia*	6	0
3342	*Streptococcus*	6	0
3531	TM7 genera incertae sedis	6	0
4246	Family *Leptotrichiaceae*	6	2
5165	*Campylobacter*	6	0
5735	*Leptotrichia*	6	1
6066	*Capnocytophaga*	6	0
6426	Family *Leptotrichiaceae*	6	0
8045	*Neisseria*	6	2

The OTUs that were detected in pre-therapy samples with more than two reads but not in post-therapy samples were defined as “disappeared OTUs.”

Only the 20 OTUs that commonly disappeared from the supragingival plaque microbiota of more than six subjects after therapy are shown.

**Figure 5:**
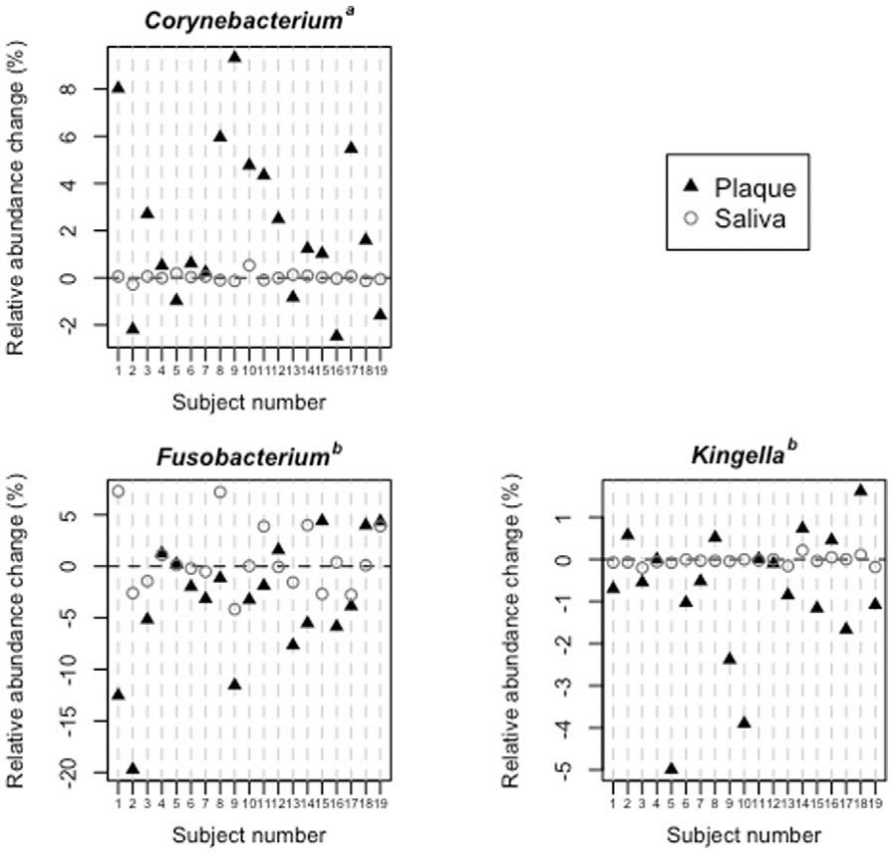
Relative abundance changes of three bacterial genera in the supragingival plaque microbiota and salivary bacterial population. ^a^ Significantly increased in the plaque microbiota after periodontal therapy. ^b^ Significantly decreased in the plaque microbiota after periodontal therapy.

Of the 92 identified genera, Fusobacterium and Kingella become significantly less predominant in plaque microbiotae after periodontal therapy, whereas the relative abundance of Corynebacterium increased (Figure 5). However, no significant changes in these genera were observed in salivary bacterial populations (Figure 5), although the abundance of four minor genera, including Granulicatella, Capnocytophaga, and Atopobium, either increased or decreased significantly (Table S3).

When reviewing at the OTU level to compare minor abundance changes in the two microbiota, of 8,497 identified OTUs, 61 and 84 became significantly more and less predominant, respectively, in the supragingival plaque and salivary microbiota after therapy (Table 6). On the other hand, the OTUs whose relative abundances significantly changed were phylogenetically different in the two bacterial communities and no OTU significantly increased or decreased in both plaque and saliva (Table 6). The OTUs whose relative abundances substantially changed following therapy in the salivary bacterial population are listed in Table S4. The relative abundance of OTUs corresponding to *Granulicatella*, *Neisseria*, and *Streptococcus* decreased in salivary bacterial populations after therapy, whereas the OTUs corresponding to bacterial genera such as *Prevotella* and *Veillonella* increased.

**Table 6:** Taxonomic classification of operational taxonomic units (OTUs) whose relative abundances in the microbiotae were significantly altered after periodontal therapy.

	Number of OTUs			
	Less predominant after therapy		More predominant after therapy	
Taxonomic classification	Plaque	Saliva	Plaque	Saliva
*Fusobacterium*	10	1	0	3
*Capnocytophaga*	6	0	0	0
*Tannerella*	5	0	0	0
*Leptotrichia*	4	0	0	7
*Neisseria*	3	3	0	0
Family *Neisseriaceae*	3	0	0	0
Family *Fusobacteriaceae*	2	0	0	0
*Kingella*	2	0	0	0
*Porphyromonas*	2	4	0	0
*Aggregatibacter*	1	0	0	0
Family *Leptotrichiaceae*	1	0	0	2
*Schlegelella*	1	0	0	2
*Rothia*	1	0	2	1
*Granulicatella*	1	10	0	0
*Streptococcus*	1	15	0	2
*Actinomyces*	1	0	3	3
Order *Actinomycetales*	1	1	2	6
*Corynebacterium*	0	0	3	0
*Prevotella*	0	3	1	4
Family *Corynebacteriaceae*	0	0	1	0
Family *Flavobacteriaceae*	0	0	1	0
Family *Propionibacteriaceae*	0	0	1	0
*Selenomonas*	0	0	1	0
*Treponema*	0	0	1	0
Domain *Bacteria*	0	3	0	0
Phylum *Firmicutes*	0	1	0	1
*Haemophilus*	0	1	0	0
*Mycoplasma*	0	1	0	0
*Pasteurella*	0	1	0	0
Family *Prevotellaceae*	0	1	0	0
*Veillonella*	0	1	0	2
*Atopobium*	0	0	0	1
Order *Clostridiales*	0	0	0	1
Order *Lactobacillales*	0	0	0	1
TM7 genera incertae sedis	0	0	0	2
Total number of OTUs	45	46	16	38

Significant differences in each OTU were assessed using Wilcoxon signed-rank tests. P values<0.05 were considered statistically significant.

## Discussion

Our data suggest the compositional stability of salivary bacterial populations against shifts in the supragingival plaque microbiota following periodontal therapy. UniFrac analysis revealed that the degree of microbiota shift in saliva was significantly smaller than that in supragingival plaque (Figures 2A and S1B), even when compared to interindividual differences (Figures 2B and S1C). Along with an improved periodontal condition, microbial richness and diversity significantly decreased in the supragingival plaque microbiota, whereas no such decrease occurred in the salivary bacterial population (Table 3). Although the relative abundances of some OTUs were also significantly altered in the salivary bacterial population, these were phylogenetically different from those in the supragingival plaque microbiota (Tables 6 and S4). These results suggest that the effect of the supragingival plaque microbiota on the salivary bacterial population is limited.

The patients in this study exhibited obvious clinical improvement (Table 1) after periodontal therapeutic intervention, including scaling, curettage, tooth brushing instruction, and professional mechanical tooth cleaning, but not surgical intervention or antibiotics. The shift after periodontal therapy in the supragingival plaque microbiota was smaller than the compositional difference between the two bacterial communities (Figure 1A); both the microbial richness estimated by the Chao I and ACE indices, and the biodiversity assessed by Shannon index, decreased after periodontal therapy (Table 3). This alteration in plaque microbiotae seems reasonable because most periodontal therapeutic measures are intended to remove plaque from teeth. However, note that periodontal therapy resulted in a decrease in microbial richness and diversity, suggesting that repetitive plaque debridement prevents the development of plaque microbiota. Additionally, our data suggest that the OTU corresponding to bacterial genera such as *Tannerella*, *Porphyromonas*, *Leptotrichia*, and *Capnocytophaga* commonly disappeared from the plaque microbiota following periodontal therapy (Table 5) and the genera Fusobacterium and Kingella became significantly less predominant (Figure 5). These results are reasonable considering that *Tannerella* and *Porphyromonas* species were often implicated as the causative agents of periodontitis [15]. In addition, Fusobacterium is considered to be a core bacterium for biofilm formation because it coaggregates with both early colonizing species, such as Actinomyces and Streptococcus, and late colonizers such as Porphyromonas gingivalis and Eubacterium [16]. Their dissipation or decrease might thus be implicated in periodontal improvement. However, explaining the significant increase in Corynebacterium following periodontal therapy is difficult. Further studies will be required to elucidate the mechanisms underlying generation of the complex dental plaque ecosystem following periodontal therapy.

Supragingival plaque microbiota shifts following therapy seem to be well characterized as described above, whereas pre- and post-therapy samples were not discriminated in PCoA plots based on UniFrac distance, which takes phylogeny into consideration (Figures 1A and S1A). This implies that the plaque microbiota shift did not exhibit a simple phylogenetic pattern and that relatively large shifts, other than those mentioned above, did not consistently occur throughout the sample cohort. However, summarizing all microbiota shifts is difficult because they did not exhibit significant differences. A larger sample size would be required to characterize supragingival plaque microbiota shifts resulting from periodontal therapeutic intervention in more detail.

Temporal stability of a salivary bacterial population was indicated in previous studies using T-RFLP analysis [17], [18] as well as barcoded pyrosequencing analysis [19], whereas no treatment intervention was performed in sample collection intervals. Our study further revealed that this stable bacterial community, especially its membership, was well maintained for a long period (about 2 years) accompanied by periodontal therapeutic intervention (Figures 2B and S1C). Although the stability was slightly weaker when weighted versions of the UniFrac values (also considering abundance information) were used (Figure S1C), this trend was consistent with previous results in studies without intervention [19]. Some abundance fluctuations always occur in a salivary bacterial population and were likely to be evaluated when using weighted UniFrac values. In either case, the degree of salivary microbiota shift was significantly smaller than its interindividual difference in both UniFrac analyses (Figures 2B and S1C). Dental plaque control has been suggested to produce only a small effect on the overall composition of salivary bacterial populations.

UniFrac analysis showed that the overall composition of salivary bacterial populations were well conserved after therapy (Figure 2), whereas as many as 50 OTUs disappeared and appeared after therapy in salivary bacterial populations (Tables 4). Considering low the UniFrac distance between the pre- and post-therapy samples, the disappeared OTUs and the appeared OTUs are likely to be phylogenetically close to each other. Of course, one possibility is that some turnover of related species occurs in a salivary bacterial population. However, the OTUs derived from identical bacterial species might be regarded as distinct OTUs due to insufficient accuracy of the pyrosequencing approach. Technical improvements are required to clarify this concern.

Periodontal therapy resulted in the expected rearrangement of supragingival plaque formation and likely contributed to the improvement in oral health and recovery of periodontal tissue condition. In contrast, overall salivary bacterial composition was well conserved after therapy. Although slight changes were observed at the OTU level, they were considered to be independent of the supragingival microbiota shift. These findings further emphasize that the salivary bacterial population is little affected by the supragingival plaque microbiota and reflects mostly oral bacterial communities other than dental plaque, probably the mucosal microbiota. Thus, interpreting salivary bacterial composition with caution for the purpose of evaluating dental plaque is important. Additionally, considering the previously reported impact of salivary bacterial populations on health [2], [3], [4], [5], [6], an effective oral mucosal microbiota treatment other than teeth cleaning is needed to improve and maintain health.

## Materials and Methods

### Ethics statement

Written informed consent was obtained from all participants. The ethics committee of Kyushu University Faculty of Dental Science approved this study and the procedure for obtaining informed consent.

### Subjects and clinical examinations

The study population consisted of 19 patients with periodontitis who visited the YA Dental Clinic in Yonago, Tottori, Japan. All subjects had at least 19 teeth. For each subject, the periodontal condition of all teeth was assessed following sample collection. Periodontal pocket depth at six sites (mesiobuccal, midbuccal, distobuccal, distolingual, midlingual, and mesiolingual) per tooth was measured using a periodontal pocket probe. Inclusion criteria were generally healthy adults, with no use of antibiotics or periodontal surgery during the preceding 6 months or during the periodontal therapy. Sample collection and clinical evaluations were repeated in the maintenance phase after periodontal therapy, approximately 2 years after the first sample collection.

### Sample preparation

The patients were asked to bite on paraffin wax for 5.5 min, and stimulated saliva samples produced during the final 5 min were collected in sterile plastic tubes. Sterile curettes were used to collect supragingival plaque from all tooth surfaces, in the side of the upper half-jaw that contained most teeth, using coronal strokes starting from the gingival margin. When both sides of the jaw contained equal numbers of teeth, the side to be sampled was selected randomly. Samples were stored at −30°C until further analysis. DNA extraction from each sample was performed as described previously [20].

### Barcoded pyrosequencing analysis

All 76 samples (two each from 19 subjects pre- and post-periodontal therapy) were examined using barcoded pyrosequencing analysis of the 16S rRNA gene. The 16S rRNA genes of each sample were amplified using the following primers: 338R with the 454 Life Sciences (Roche, Basel, Switzerland) adaptor B sequences (5′-CCT ATC CCC TGT GTG CCT TGG CAG TCT CAG TGC TGC CTC CCG TAG GAG T-3′) and 8F with adaptor A and subject-specific six-base barcode sequences (5′-CCA TCT CAT CCC TGC GTG TCT CCG ACT CAG NNN NNN AGA GTT TGA TYM TGG CTC AG-3′). PCR amplification was performed as described previously [21]. Amplicons were gel-purified using a Wizard SV Gel and PCR Clean-Up System (Promega, Madison, WI) according to the manufacturer's instructions. DNA concentration and quality were assessed using a NanoDrop spectrophotometer (NanoDrop Technologies, Wilmington, DE), and equal amounts of DNA from 38 supragingival plaque and saliva samples were pooled. Pyrosequencing was conducted using a 454 Life Sciences Genome Sequencer FLX instrument (Roche) at Hokkaido System Science Co., Ltd. (Sapporo, Japan).

### Data analysis and taxonomy assignment

Sequences were excluded from the analysis using a script written in PHP, if they were shorter than 240 bases or had an average quality score <25, and subsequently removed using a script written in R if they did not include the correct primer sequence, had a homopolymer run >6 nt, or contained ambiguous characters. The remaining sequences were assigned to the appropriate subject by examining the six-base barcode sequence. Similar sequences were clustered into OTUs using UCLUST [22], with a minimum pairwise identity of 97%. Seed sequences from each OTU were aligned using PyNAST [23] and the Greengenes database [24] using a minimum identity of 75%. Chimeras were removed from the representative set on the basis of identification as chimeric via Chimera Slayer [25] and verification that the putative chimera appeared in only one sample. After chimera elimination, a relaxed neighbor-joining tree was built using FastTree [26]. The UniFrac metric [27] calculated by FastUnifrac [28] was used to determine any dissimilarity between pairs of bacterial communities. UniFrac distances were based on the fraction of branch length shared between two communities, within a phylogenetic tree constructed from all communities being compared. The similarity relationship assessed using the unweighted UniFrac metric was represented in a PCoA plot drawn by R. The taxonomy of representative sequences was determined using the RDP classifier with a minimum support threshold of 60% and the RDP taxonomic nomenclature (down to genus level).

### Statistical analysis

All statistical analyses were conducted with R 2.13.2 [29]. OTU numbers, Chao I index, and ACE index were calculated using the Vegan package in R. Paired t-tests were performed to compare UniFrac distances between pre- and post-therapy samples, the number of OTUs, microbial richness, diversity, and the relative abundances of each phylum and genus. Student's t-tests were performed to compare the combined pre- and post-therapy UniFrac distances for the same individual with those of the 18 other individuals combined. Wilcoxon signed-rank tests were conducted to compare the relative abundances of each OTU.

## Supporting Information

Figure S1 **Weighted UniFrac analysis.** (A) Principal coordinate analysis (PCoA) plot of similarity relations among the 76 bacterial community samples. Plots were generated using the weighted UniFrac metric. These two components explain 56.2% of the variance. (B) Weighted UniFrac distance between pre- and post-therapy samples. Significant differences between the supragingival plaque microbiota and salivary bacterial population were assessed using paired t-tests. ***P<0.001. (C) Weighted UniFrac distance between pre-and post-therapy saliva samples. UniFrac distance of the combination of pre- and post-therapy for the same individual (•) and those of the other 18 individuals (×) were plotted for each subject. A significant difference was observed between the UniFrac distance of the combination of pre- and post-therapy for the same individual and those of the other 18 individuals by Student's *t*-test (*P* = 0.002).
(TIF)

Figure S2 **Mean phylum abundances in the supragingival plaque microbiota and salivary bacterial population before and after periodontal therapy.**
(TIF)

Table S1 **Relative abundance of each phylum in the salivary bacterial population and supragingival microbiota.**
(DOCX)

Table S2 **Relative abundance of each genus in the salivary bacterial population and supragingival microbiota.**
(DOCX)

Table S3 **Changes in relative abundance of individual genera in the salivary bacterial population.**
(DOCX)

Table S4 **Changes in relative abundance of operational taxonomic units (OTUs) whose relative abundances in the microbiota were significantly altered after periodontal therapy.**
(DOCX)
