# Supplementary figures and images for: Compositional Stability of a Salivary Bacterial Population against Supragingival Microbiota Shift following Periodontal Therapy
Source: PLoS One. 2012 Aug 16;7(8):e42806. doi: 10.1371/journal.pone.0042806 (PMC3420916; doi:10.1371/journal.pone.0042806)

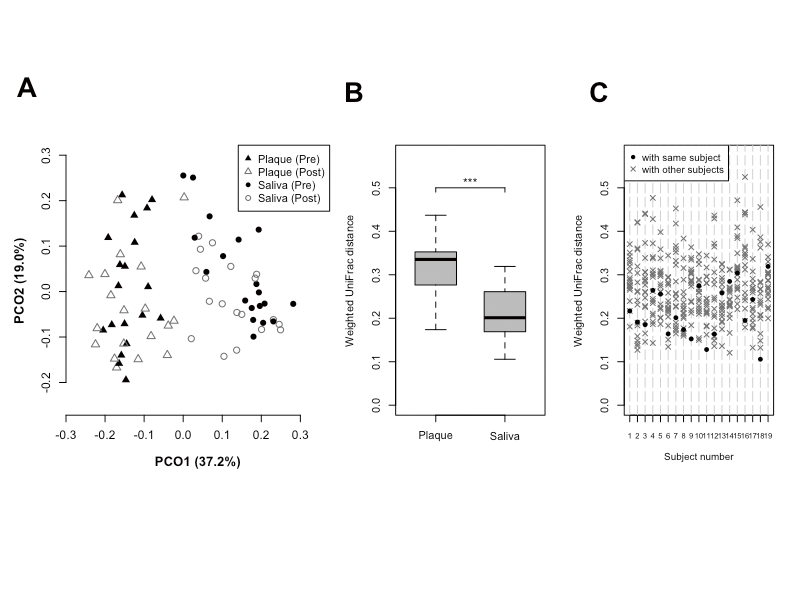

Supplement: Figure S1 — Weighted UniFrac analysis. (A) Principal coordinate analysis (PCoA) plot of similarity relations among the 76 bacterial community samples. Plots were generated using the weighted UniFrac metric. These two components explain 56.2% of the variance. (B) Weighted UniFrac distance between pre- and post-therapy samples. Significant differences between the supragingival plaque microbiota and salivary bacterial population were assessed using paired t-tests. ***P<0.001. (C) Weighted UniFrac distance between pre-and post-therapy saliva samples. UniFrac distance of the combination of pre- and post-therapy for the same individual (•) and those of the other 18 individuals (×) were plotted for each subject. A significant difference was observed between the UniFrac distance of the combination of pre- and post-therapy for the same individual and those of the other 18 individuals by Student's t-test (P = 0.002). (TIF) [file pone.0042806.s001.tif]

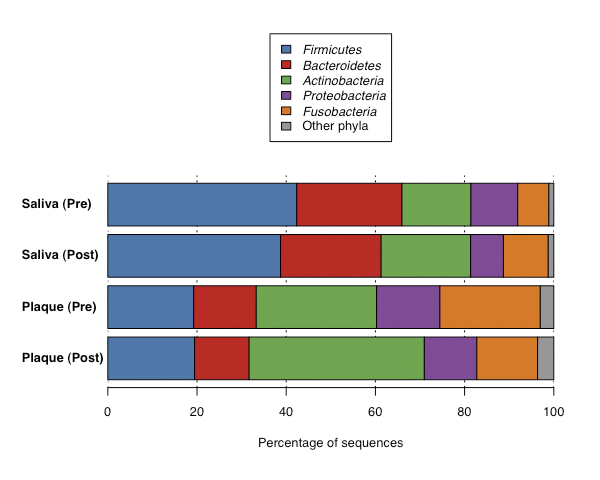

Supplement: Figure S2 — Mean phylum abundances in the supragingival plaque microbiota and salivary bacterial population before and after periodontal therapy. (TIF) [file pone.0042806.s002.tif]
