# Supplementary material for: Compositional Stability of a Salivary Bacterial Population against Supragingival Microbiota Shift following Periodontal Therapy
Source: PLoS One. 2012 Aug 16;7(8):e42806. doi: 10.1371/journal.pone.0042806 (PMC3420916; doi:10.1371/journal.pone.0042806)
Supplement: Table S1 — Relative abundance of each phylum in the salivary bacterial population and supragingival microbiota. (DOCX) [file pone.0042806.s003.docx]

**Table S1.** Relative abundance of each phylum in the salivary bacterial population and supragingival microbiota.

|  | Relative abundance (%) | |  |
| --- | --- | --- | --- |
|  | Saliva | Supragingival plaque | *P* value |
| *Firmicutes* | 40.5 ± 10.1 | 19.3 ± 9.4 | **<0.001** |
| *Bacteroidetes* | 23.0 ± 8.6 | 13.1 ± 6.6 | **<0.001** |
| *Actinobacteria* | 17.7 ± 6.5 | 33.1 ± 18.4 | **<0.001** |
| *Proteobacteria* | 8.9 ± 8.0 | 12.9 ± 10.8 | **0.012** |
| *Fusobacteria* | 8.5 ± 5.4 | 18.0 ± 10.9 | **<0.001** |
| TM7 | 0.5 ± 0.6 | 1.31 ± 2.68 | 0.070 |
| *Spirochaetes* | 0.07 ± 0.15 | 0.45 ± 0.82 | **0.003** |
| SR1 | 0.02 ± 0.06 | 0.01 ± 0.03 | 0.230 |
| *Tenericutes* | 0.01 ± 0.02 | 0.00 ± 0.01 | **0.015** |
| *Synergistetes* | 0.01 ± 0.03 | 0 | **0.041** |
| *Cyanobacteria* | 0.01 ± 0.02 | 0 | 0.074 |

Significant difference was assessed by paired t test. *P* values < 0.05 were shown in bold.
