# Supplementary material for: Compositional Stability of a Salivary Bacterial Population against Supragingival Microbiota Shift following Periodontal Therapy
Source: PLoS One. 2012 Aug 16;7(8):e42806. doi: 10.1371/journal.pone.0042806 (PMC3420916; doi:10.1371/journal.pone.0042806)
Supplement: Table S2 — Relative abundance of each genus in the salivary bacterial population and supragingival microbiota. (DOCX) [file pone.0042806.s004.docx]

**Table S2.** Relative abundance of each genus in the salivary bacterial population and supragingival microbiota.

|  | Relative abundance (%) | |  |
| --- | --- | --- | --- |
|  | Saliva | Supragingival plaque | *P* value |
| Significantly more predominant in saliva | | | |
| *Streptococcus* | 21.3 ± 8.7 | 10.9 ± 8.0 | <0.001 |
| *Prevotella* | 17.4 ± 9.2 | 2.9 ± 2.6 | <0.001 |
| *Veillonella* | 8.4 ± 4.0 | 2.2 ± 2.7 | <0.001 |
| *Gemella* | 2.8 ± 2.8 | 0.5 ± 0.8 | <0.001 |
| *Granulicatella* | 2.2 ± 1.4 | 0.6 ± 1.1 | <0.001 |
| *Haemophilus* | 0.5 ± 0.6 | 0.06 ± 0.19 | <0.001 |
| *Megasphaera* | 0.3 ± 0.3 | 0.02 ± 0.04 | <0.001 |
| *Solobacterium* | 0.2 ± 0.1 | 0.03 ± 0.06 | <0.001 |
| *Atopobium* | 0.1 ± 0.1 | 0.05 ± 0.13 | <0.001 |
| *Oribacterium* | 0.1 ± 0.1 | 0.05 ± 0.10 | 0.001 |
| *Moryella* | 0.06 ± 0.07 | 0.03 ± 0.04 | 0.014 |
| *Streptobacillus* | 0.06 ± 0.15 | 0.01 ± 0.02 | 0.036 |
| *Mycoplasma* | 0.01 ± 0.02 | 0.00 ± 0.01 | 0.016 |
| *Pyramidobacter* | 0.01 ± 0.03 | 0 | 0.041 |
| *Pasteurella* | 0.01 ± 0.02 | 0 | 0.027 |
| *Staphylococcus* | 0.00 ± 0.01 | 0 | 0.045 |
| Significantly more predominant in supragingival plaque | | | |
| *Actinomyces* | 6.9 ± 4.8 | 11.3 ± 11.2 | 0.032 |
| *Leptotrichia* | 3.7 ± 3.2 | 11.0 ± 7.8 | <0.001 |
| *Capnocytophaga* | 0.2 ± 0.3 | 4.8 ± 3.3 | <0.001 |
| *Corynebacterium* | 0.09 ± 0.12 | 3.7 ± 3.1 | <0.001 |
| *Schlegelella* | 0.3 ± 0.6 | 2.4 ± 3.4 | <0.001 |
| *Selenomonas* | 0.1 ± 0.0 | 1.6 ± 1.3 | <0.001 |
| *Tannerella* | 0.1 ± 0.2 | 1.5 ± 2.8 | 0.004 |
| *Campylobacter* | 0.3 ± 0.2 | 1.1 ± 0.9 | <0.001 |
| *Kingella* | 0.07 ± 0.07 | 1.0 ± 1.1 | <0.001 |
| *Cardiobacterium* | 0.03 ± 0.04 | 0.7 ± 0.7 | <0.001 |
| *Ottowia* | 0.01 ± 0.04 | 0.5 ± 1.4 | 0.033 |
| *Treponema* | 0.07 ± 0.15 | 0.4 ± 0.8 | 0.003 |
| *Schwartzia* | 0.01 ± 0.01 | 0.1 ± 0.1 | 0.002 |
| *Dialister* | 0.06 ± 0.06 | 0.1 ± 0.2 | 0.011 |
| *Centipeda* | 0.01 ± 0.02 | 0.09 ± 0.18 | 0.006 |
| *Elizabethkingia* | 0.01 ± 0.02 | 0.04 ± 0.06 | <0.001 |
| *Weeksella* | 0.00 ± 0.01 | 0.03 ± 0.07 | 0.009 |
| *Turicella* | 0 | 0.03 ± 0.09 | 0.038 |
| *Propionivibrio* | 0.00 ± 0.01 | 0.02 ± 0.04 | 0.014 |
| *Johnsonella* | 0 | 0.02 ± 0.04 | 0.009 |
| *Zhouia* | 0 | 0.02 ± 0.05 | 0.024 |
| *Howardella* | 0 | 0.00 ± 0.01 | 0.029 |

Only 38 genera that exhibited significant differences between the salivary bacterial population and supragingival microbiota are shown. Significant differences were assessed using paired t-tests.
