# Supplementary material for: Compositional Stability of a Salivary Bacterial Population against Supragingival Microbiota Shift following Periodontal Therapy
Source: PLoS One. 2012 Aug 16;7(8):e42806. doi: 10.1371/journal.pone.0042806 (PMC3420916; doi:10.1371/journal.pone.0042806)
Supplement: Table S3 — Changes in relative abundance of individual genera in the salivary bacterial population. (DOCX) [file pone.0042806.s005.docx]

**Table S3.** Changes in the relative abundance of individual genera in the salivary bacterial population.

|  | Relative abundance (%) | |  |
| --- | --- | --- | --- |
|  | Pre-therapy | Post-therapy | *P* value |
| *Streptococcus* | 22.9 ± 6.5 | 19.7 ± 10.4 | 0.207 |
| *Prevotella* | 17.0 ± 9.7 | 17.7 ± 9.1 | 0.676 |
| *Veillonella* | 7.7 ± 4.0 | 9.2 ± 3.9 | 0.218 |
| *Rothia* | 7.4 ± 4.5 | 8.6 ± 5.6 | 0.494 |
| *Actinomyces* | 5.8 ± 3.8 | 8.0 ± 5.5 | 0.142 |
| *Neisseria* | 5.7 ± 6.2 | 3.6 ± 4.2 | 0.185 |
| *Porphyromonas* | 5.3 ± 7.1 | 3.7 ± 5.9 | 0.262 |
| *Leptotrichia* | 2.9 ± 2.7 | 4.5 ± 3.4 | 0.117 |
| *Fusobacterium* | 3.1 ± 2.2 | 3.7 ± 3.9 | 0.402 |
| *Gemella* | 3.2 ± 3.1 | 2.4 ± 2.6 | 0.139 |
| *Granulicatella* | 2.8 ± 1.4 | 1.7 ± 1.1 | **0.005** |
| *Haemophilus* | 0.6 ± 0.8 | 0.5 ± 0.4 | 0.328 |
| TM7 | 0.3 ± 0.3 | 0.6 ± 0.8 | 0.067 |
| *Schlegelella* | 0.2 ± 0.3 | 0.5 ± 0.8 | 0.191 |
| *Megasphaera* | 0.3 ± 0.3 | 0.3 ± 0.3 | 0.526 |
| *Campylobacter* | 0.3 ± 0.2 | 0.3 ± 0.2 | 0.480 |
| *Capnocytophaga* | 0.3 ± 0.4 | 0.2 ± 0.3 | **0.031** |
| *Solobacterium* | 0.2 ± 0.1 | 0.2 ± 0.2 | 0.650 |
| *Lactobacillus* | 0.1 ± 0.2 | 0.2 ± 1.1 | 0.624 |
| *Atopobium* | 0.1 ± 0.0 | 0.2 ± 0.2 | **0.017** |
| *Peptostreptococcus* | 0.2 ± 0.4 | 0.09 ± 0.11 | 0.070 |
| *Tannerella* | 0.1 ± 0.1 | 0.2 ± 0.2 | 0.402 |
| *Parvimonas* | 0.1 ± 0.1 | 0.1 ± 0.1 | 0.722 |
| *Oribacterium* | 0.1 ± 0.09 | 0.1 ± 0.1 | 0.373 |
| *Abiotrophia* | 0.1 ± 0.2 | 0.1 ± 0.2 | 0.395 |

Only the 25 most dominant bacterial genera (relative proportion >0.1%) in salivary bacterial populations are listed. Significant differences were assessed using paired t-tests. P values <0.05 are indicated in bold type.
