# Supplementary material for: Compositional Stability of a Salivary Bacterial Population against Supragingival Microbiota Shift following Periodontal Therapy
Source: PLoS One. 2012 Aug 16;7(8):e42806. doi: 10.1371/journal.pone.0042806 (PMC3420916; doi:10.1371/journal.pone.0042806)
Supplement: Table S4 — Changes in relative abundance of operational taxonomic units (OTUs) whose relative abundances in the microbiota were significantly altered after periodontal therapy. (DOCX) [file pone.0042806.s006.docx]

**Table S4.** Changes in relative abundance of operational taxonomic units (OTUs) whose relative abundances in the microbiotae were significantly altered after periodontal therapy.

|  | Relative abundance (%, median) | |  |
| --- | --- | --- | --- |
| OTU number | Pre-therapy | Post-therapy | Taxonomic information |
| Significantly decreased in saliva | | | |
| 3914 | 0.68 | 0.30 | *Granulicatella* |
| 7939 | 0.93 | 0.62 | *Neisseria* |
| 6732 | 0.43 | 0.25 | *Streptococcus* |
| 4136 | 0.34 | 0.18 | *Granulicatella* |
| 6975 | 0.23 | 0.09 | *Granulicatella* |
| Significantly decreased in supragingival plaque | | | |
| 109 | 2.16 | 0.7 | *Fusobacterium* |
| 7452 | 0.55 | 0.03 | *Capnocytophaga* |
| 834 | 0.38 | 0.07 | *Fusobacterium* |
| 2898 | 0.25 | 0.00 | *Tannerella* |
| 2104 | 0.28 | 0.15 | *Kingella* |
| Significantly increased in saliva | | | |
| 4792 | 0.2 | 0.56 | *Prevotella* |
| 4184 | 0.13 | 0.29 | *Veillonella* |
| 7478 | 0.03 | 0.19 | Family *Leptotichiaceae* |
| 7456 | 0.04 | 0.19 | *Leptotrichia* |
| 6431 | 1.22 | 1.36 | *Veillonella* |
| 8110 | 0.13 | 0.26 | *Actinomyces* |
| Significantly increased in supragingival plaque | | | |
| 2353 | 0.66 | 1.16 | *Corynebacterium* |
| 1949 | 0.08 | 0.24 | Family *Corynebateriaceae* |
| 2714 | 0.05 | 0.21 | *Actinomyces* |

Only 19 OTUs whose relative abundances in the microbiotae were greatly altered (relative abundance shift > 0.1% in median) after periodontal therapy were shown.
